# Adolescents in the Covid Net: What Impact on their Mental Health?

**DOI:** 10.1007/s40653-022-00497-8

**Published:** 2022-11-23

**Authors:** Chiara Ionio, Giulia Ciuffo, Federica Villa, Marta Landoni, Maddalena Sacchi, Damiano Rizzi

**Affiliations:** 1 CRIdee, Trauma Research Unit, Dipartimento Di Psicologia, Università Cattolica, Largo Gemelli 1, 20123 Milan, Italy; 2 Fondazione Soleterre Onlus, c/o Abbazia Di Mirasole, Strada Consortile del Mirasole, 7, Opera (MI), Italy

**Keywords:** Adolescence, Youth, Covid-19, Psychological well-being, Trauma, Emotional regulation

## Abstract

Given the impact of traumatic events in adolescence and early adulthood, the current Covid 19 pandemic poses a high risk to the well-being and mental health of this population. This study aims to shed light on the traumatic impact of Covid-19 on adolescents and young adults, taking into account their personal experiences, with a particular focus on their emotional regulation skills. From May 2021 to May 2022, 216 adolescents and young adults were surveyed using a series of self-report questionnaires to assess the potentially traumatic effects of Covid-19 and its impact on adolescents' and young adults' emotional regulation skills. Analyses revealed a significant traumatic effect of Covid-19 on the adolescents in our sample. Significant correlations also emerged between the impact of the traumatic event and adolescents' emotional regulation skills. Our findings underscore the importance of considering the psychological impact of Covid-19 on adolescents from both a restorative and preventive perspective.

## Introduction

Trauma is a psychological reaction triggered by a traumatic event that consists of being overwhelmed by distressing and unbearable feelings (Caretti & Craparo, 2008). Among the mechanisms underlying trauma is the failure to explicitly process and integrate the intense emotional experiences of the traumatic event into autobiographical memory (see, e.g., Brewin, 2001; Bucci, 1997; Liotti, 2004; Brown & Kulik, 1977).

Moreover, traumatic experiences have particular resonance during adolescence and pre-adulthood (Ammaniti et al., 2008; Ionio et al., 2013). Adolescence is a transitional period characterised by numerous dramatic intraindividual changes at physical, cognitive, and emotional levels (Sewell & Cruise, 2009). It is therefore a critical developmental period that can influence the likelihood, severity, and course of later mental health problems (Kessler et al., 2007).

For adolescents and youths in particular, trauma exposure leads to a negative redefinition of behaviour, emotions, and cognitive processes because their ability to regulate emotions and process information is disrupted (Ardino, 2009). Indeed, traumatic experiences in adolescence and pre-adulthood can lead to significant changes in brain regions and systems that play critical roles in behaviour regulation, emotion regulation, and risk and hazard assessment (Ammaniti et al., 2008; Steinberg, 2005). The literature has shown that adolescents and youths are particularly vulnerable to prolonged stress because the brain regions that regulate the stress response, such as the pituitary, hippocampus, amygdala, and prefrontal cortex, are still developing at this stage of development (Romeo, 2013).

In light of this, and given that the current pandemic situation is associated with lower mental well-being even in the general population than in the previous period (Vindegaard & Benros, 2020), a new, highly topical, and unfortunately still relevant issue concerns the potential impact of COVID -19 and related events on adolescent and youths mental health in general and from a trauma perspective in particular (see Octavius et al., 2020; Imran et al., 2020). Given the constant risk of COVID -19 harming oneself or loved ones, as well as the other emotional, mental, and financial burdens of the pandemic, it is clear that the pandemic may be a chronic traumatic stressor for many adolescents and youths, particularly for the Italian population.

Italy was, in fact, the first Western country to be severely affected by COVID-19, with the highest number of infected for many weeks (Fiorillo et al., 2020). At that time, on March 8, 2020, the Italian Prime Minister imposed a curfew for the entire population until May 3, 2020. With higher mortality than previously observed in China (7.2 vs. 2.3%), the epidemic took a more severe course in Italy (Fiorillo et al., 2020).

During this time, the task of parenting took on even more facets: parents felt more pressure to work from home while caring for school-aged children at home, without support from other family members or other social support networks (Cluver et al., 2020; Fegert et al., 2020; Mensi et al., 2021). In particular, when family members have died, relationships and support systems can become strained, leading to the development of PTSD in both parents and their children (Mensi et al., 2021). For adolescents and youths in particular, the emotional stress of parents can exacerbate their symptoms if they have been overwhelmed by the crisis (COVID -19), resulting in tremendous stress and psychological distress (Mensi et al., 2021; Wagner, 2020)In addition, this was a particularly difficult time for teens and youths who were underprivileged or marginalized, such as ones with a history of trauma (Fegert et al., 2020), who tend to have higher levels of psychological distress (Duan et al., 2020).

In light of this, it is safe to say that the pandemic could be traumatizing for the general population (Horesh & Brown, 2020) and is an independent factor causing post-traumatic stress symptoms in adolescents and young adults d (Ma et al., 2021). Therefore, some recent research and reviews have focused on examining the prevalence of post traumatic symptoms in adolescents in COVID -19 and the pandemic situation.

For example, Panda et al. (2020) found that 91% of adolescents were anxious about the COVID -19 pandemic; Ma et al. (2021) demonstrated a 48% prevalence of posttraumatic stress symptoms related to COVID -19, and Mensi et al. (2021) demonstrated the presence of ASD (acute stress disorder) or PTSD (posttraumatic stress disorder) in 79.52% of adolescents.

The Italian research landscape also provided some interesting results on the impact of COVID -19 on the adolescent population, focusing on the stressful and traumatic aspects of COVID -19 among Italian adolescents. Buzzi et al. (2020) found that 65.7% of Italian adolescents were moderately to severely concerned about the COVID -19 pandemic, while Mensi et al. (2021) showed a prevalence of ASD and PTSD symptoms of 29.48% and 50.04%, respectively, among Italian adolescents.

Particular attention should be paid to trauma and difficulties in emotion regulation, as little research has been conducted on how trauma and PTSD affect impulsivity and dysregulation (Ardino, 2009). The literature now describes that PTSD in minors is associated with changes in the frontal limbic circuitry that may contribute to increased reactivity to threatening stimuli and weaker emotion regulation (Guessoum et al., 2020; Herringa, 2017).

Adolescents and youths may therefore be an important target for this type of intervention. The developmental phase of adolescence and youthhoods are characterized by maturational changes in emotion regulation, which in turn influence vulnerability to psychopathology.

Following trauma exposure, the adolescent and youth's ability to regulate emotions and process information are impaired (Ardino, 2009). Villalta et al. (2018), indeed, confirmed that available research suggests a strong link between difficulties in emotion regulation and posttraumatic stress symptoms in adolescents and youths.

Although these studies highlight the importance of trauma exposure, few studies have examined whether the effects of trauma exposure on emotion dysregulation differ depending on the timing of the event in development. Thus, the existence of "sensitive periods" (Dunn et al., 2013) for the occurrence of emotion dysregulation, or windows of time when the developing human brain is particularly vulnerable or sensitive to trauma and when trauma exposure consequently leads to higher levels of emotion dysregulation, is still unknown.

It is also conceivable that the timing of trauma exposure during the developmental years from infancy to adolescence has nothing to do with emotion dysregulation or varies depending on the nature of the trauma.

Differentiating basic emotions in preschool children, elaborating on emotional expression in school-aged children, and understanding the causes and effects of negative emotions in adolescents have shown that social adversity disrupts the maturational mechanisms of emotion regulation at different developmental stages (Pynoos et al., 1999). These findings suggest that there may be different developmental stages of emotion regulation that are particularly vulnerable to adversity during childhood and adolescence, possibly depending on the type of trauma exposure. These interactions may have different, detrimental effects on future emotion dysregulation.

Given these risks and the fact that COVID -19 has also been defined as a potentially traumatic event for adolescents and youths (Ma et al., 2021), given the highlighted relationship between the two variables of interest in this section, it is necessary to ask about the relationship between the traumatic impact of COVID -19 and emotion regulation problems in adolescents and young adults: the relationship between trauma and emotion regulation difficulties has been little studied in adolescents without a previous history of trauma and in young adults in general (Villalta et al., 2018). Understanding comprehensively of how young people respond to this currently abnormal and pervasive phenomenon could help mitigate its potential and long-term negative effects.

Therefore, the present work aims to assess the traumatic effects of Covid-19 on adolescents and young adults, taking into account their personal experiences and paying particular attention to their emotional regulation abilities. We hypothesized that there would be an association between the traumatic effects of Covid-19 and personal historical events during the pandemic. We expected that the traumatic impact of Covid-19 would be greater among adolescents and youths who tested positive for Covid or were hospitalized, or among adolescents and youths who witnessed the positivity and/or hospitalization of a relative or friend. Finally, we hypothesized that greater traumatic impact of Covid-19 would be associated with greater difficulties in emotion regulation.

## Materials and Methods

### Data Collection

This study is part of a larger research effort conducted by the Trauma Psychology Research Unit (CRIdee) at the Catholic University of the Sacred Heart and sponsored by the Soleterre Onlus Foundation to examine the impact of COVID-19 on adolescents and young adults as a traumatic event. The purpose of this study was to explore the impact of the containment measures taken after the spread of Covid-19, and thus to examine how adolescents and young adults experienced the pandemic and how they responded emotionally.

The research was publicised through formal channels such as schools and informal channels based on word of mouth among relatives and friends. Minor participants were required to provide informed consent signed by both parents. Adult participants were asked to sign the same consent form.

Participants were provided a link to the Qualtrics platform to access the survey. All data collected were entered into a dedicated database from May 2021 to May 2022, where participants were identified only by a unique ID number. The database was stored on a secure server, and access to the information was limited to members of the research team.

### Participants

Our sample consisted of 216 adolescents and young adults aged 12 to 23 years (17.23 ± 2.041) as defined by Sawyer et al. (2018). It was composed of 54% males and 46% females. 97.2% of the sample were Italian adolescents, most of whom (75.9%) attended secondary school. 84.3% of the adolescents reported having siblings. 56.9% of the sample spent the quarantine with parents and siblings. Exclusion criteria were lack of Italian language skills and not belonging to the indicated age group (12–23 years).

### Measures

Adolescents and youths were asked to answer several multiple-choice questions designed to explore some aspects related to curfew: Relationships, school, daily life (e.g., "*Who did you spend the lockout period with?"; "Did your relationship with your friends change during the lockout period?"; "Did a relative or friend of yours test positive for covid?"*).

No screening for pre-existing trauma was conducted. In addition, they were asked to fill in the following measures:

*The Centrality of Events Scale (CES; Berntsen & Rubin,* 2006*, Italian validation by Ionio et al.,* 2018*)*

The CES is a self-report instrument designed to assess the extent to which the memory of a stressful and traumatic event was central to (a) life history, (b) personal identity, and (c) attribution of significance to other personal life events. These three factors are assessed using 20 items on a 5-point Likert scale ranging from complete disagreement to agreement. The questionnaire has good internal consistency (Cronbach's alpha = 0.94).

*Difficulty in Emotion Regulation Scale (DERS; Sighinolfi et al.,* 2010 *)*

The DERS is a 36-item self-report instrument designed to examine individual differences in the ability to recognise, accept, and cope with emotional experiences. We used the 20-item version of the scale, which consists of 5 subscales (nonacceptance, awareness, goals, clarity, impulse) and the total score. The scale showed good internal consistency (Cronbach's alpha = 0.89).

*Impact of Event Scale (IES; Weiss & Marmar,* 1997*)*

The IES-R examines the emotions people experience immediately after a potentially stressful event. It consists of 22 items divided into three clusters related to symptoms of PTSD. A third scale provides the total score, which is calculated by adding the scores from the previous rankings. The scale has good internal consistency (Cronbach's alpha = 0.69).

### Analysis

SPSS Statistics version 27.0 was used to analyze the data. Descriptive analyses were first conducted to examine the sociodemographic characteristics of the sample and to assess the situation of adolescents with the above instruments. Pearson correlations and univariate ANOVA were used to examine the influence of some sociodemographic characteristics on adolescents’ well-being and perceived trauma.

## Results

### Traumatic Impact of Covid-19 on Adolescents Young Adults

The results of the descriptive analysis performed with the IES-R showed that 8.3% of the sample fell within the range of clinical concern for PTSD, 3.3% fell within the correct clinical range for PTSD, and 28.4% fell within the range of severe PTSD. Thus, overall, 31.7% of adolescents reported that the traumatic effects of COVID-19 were clinical or severe for PTSD. Specifically, 36.7% of the sample reported high levels of avoidance symptoms related to the traumatic event, 29.1% of subjects reported high levels of intrusive symptoms, and 35.5% of participants reported high levels of hyperarousal symptoms. Results from the CES scale showed that 32.4% of adolescents reported a significant impact of traumatic memories on their daily lives. Specifically, 27.8% of the sample indicated that traumatic memories had a large impact on meaning making of their experiences, and 4.9% indicated a very large impact. 28.2% indicated that traumatic memories had a strong influence on constructing their identity, and 3.5% indicated a very strong influence. Finally, 18.6% indicated that traumatic memories had a strong influence, and 4.6% a very strong influence on their life perspectives.

The presence of a relative hospitalized for Covid-19 was found to be negatively correlated with the significance of traumatic memories (r =—0.144; p < 0.05). The results of the univariate ANOVA also showed that the presence of a Covid-positive relative had a significant impact on the hyperarousal mechanisms F (1, 214) = 4.956; p = 0.027 and on the IES total scale F (1, 214) = 4.427; p = 0.037. In addition, the presence of a covid-positive relative was found to affect the adolescent's identity construction F (1,214) = 4.305; p = 0.039, the impact of traumatic memories on life perspectives F (1, 214) = 4.324; p = 0.039, and the CES total scale (centrality of the traumatic event) F (1,214) = 4.481; p = 0.035. There were no significant results regarding a possible association between these variables and positivity and/or hospitalisation of the adolescents themselves.

### Impact of the Traumatic Event on Adolescents and Young Adults' Emotional Regulation Skills

The descriptive analysis of the DERS revealed that 20.8% of adolescents reported only moderate acceptance of emotional responses (scores between 16 and 20), 49.1% reported only moderate emotional awareness (scores between 13 and 16), 14.8% reported having only adequate competence in goal-directed behaviour (scores ranging from 13 to 16), 14.4% reported having adequate levels of emotional clarity (scores ranging from 10 to 12), while 16.7% felt they had poor impulse control (scores ranging from 13 to 16).

Table 1 summarizes some significant correlations we found between the DERS and IES dimensions. As can be seen, there were significant positive correlations between all subscales of the instruments, except for the DERS scales Awareness and Avoidance, Hyperarousal and the Total scale.

**Table 1:** Pearson correlations between DERS dimensions and IES dimensions, p < 0.01

		**IES_Avoidance**	**IES_Intrusion**	**IES_Hyperarousal**	**IES_Total**
DERS_Nonacceptance	**r p**	0.511 0.01	0.520 0.01	0.543 0.01	0.546 0.01
DERS_Awareness	**r p**	**-**	0.186 0.01	**-**	**-**
DERS_Goals	**r p**	0.516 0.01	0.563 0.01	0.603 0.01	0.584 0.01
DERS_Clarity	**r p**	0.472 0.01	0.500 0.01	0.581 0.01	0.537 0.01
DERS_Impulse	**r p**	0.536 0.01	0.574 0.01	0.587 0.01	0.589 0.01

## Discussion

### Traumatic Impact of Covid-19 on Adolescents and Young Adults

The aim of the present work was to investigate the possible traumatic impact of the Covid-19 pandemic on adolescents and young adults, and the impact on their emotional regulation abilities. The results of the analyses conducted on our sample are consistent with findings in the recent literature. A significant proportion of the sample exhibited high levels of all posttraumatic symptoms belonging to the clusters required for a PTSD diagnosis (intrusion, avoidance and hyperarousal) (APA, 2014). This first empirical finding confirms what has been analysed at the theoretical level, namely that the health burden of COVID-19 and its direct consequences are traumatic events for the general population (Bridgland et al., 2021; Horesh & Brown, 2020) and also for adolescents (Ma et al., 2021; Mensi et al., 2021). For example, Sprang and Silman (2013) found that PTSD was present in 30% of children exposed to confinement measures such as isolation or quarantine.

The association between the traumatic effects of Covid-19 and viral positivity of a relative or friend was confirmed both on the overall IES-R scale and specifically for hyperarousal symptoms. This finding is particularly interesting because all of the variables considered in the research hypotheses are consistent with the trauma definition of Criterion A for PTSD diagnosis ( i.e. death or threat of death, either by directly experiencing, directly witnessing or co-experiencing a traumatic event that happened to others or by experiencing a traumatic event that happened to a family member or friend; APA, 2014). However, only the presence of a virus-positive relative has been shown to be associated with the traumatic effects of COVID-19 for adolescents. One possible explanation lies in the fact that adolescents and young adults are at lower risk of contracting the virus and developing serious consequences if they test positive (Imran et al., 2020). This awareness may have prevented the adolescents and youths from feeling threatened, which would explain the lack of significance of the correlation between these variables. Regarding positivity of a relative, the significant correlation with traumatic effects of COVID-19 in general and with hyperarousal symptoms is consistent with what Bridgland et al. (2021) pointed out, namely that COVID-19 is also a traumatic stressor due to the emotions triggered by the negative events anticipated by the subject. Thus, news of a relative’s or friend’s positive development might have triggered a series of worries about the loved one’s safety that could have physiologically activated and frightened adolescents and young adults in a traumatic sense.

In addition, our results show that although COVID-19 was not central, as originally hypothesized, this event was important to the lives and identities of adolescents and young adults who also reported high levels of traumatic impact. This is consistent with previous findings by Ionio et al. (2018).

### Impact of the Traumatic Event on Adolescents and Young Adults' Emotional Regulation Skills

Regarding descriptive data on difficulties in emotion regulation, it should be noted that the proportions of the sample reporting difficulties in the various subscales of the DERS are generally not particularly high, with the exception of difficulties related to emotional awareness, which were rated as poor or moderate in about half of the sample. Of particular relevance to the present work, however, is not so much the prevalence of emotion regulation difficulties, but their association with the traumatic effects of COVID-19. This correlational hypothesis was largely confirmed. Indeed, the subscales of the DERS related to difficulties in accepting emotions, adopting goal-directed behaviour, emotional clarity and impulse control correlated with all subscales of the IES-R, including the total scale, whereas difficulties in emotional awareness correlated only with the intrusion symptoms subscale of the IES-R's. These results confirm the findings of Paschke et al. (2021), who found that, in adolescents, lockdown due to COVID-19 led to a significant increase in psychological distress and emotional dysregulation. Our findings also confirm the relationship between trauma and difficulties in emotion regulation, which has been widely supported theoretically (Caretti & Craparo, 2008; Dunn et al., 2018; Tull et al., 2007) and, more importantly, add to the body of knowledge on this topic, the lack of which has been pointed out (Tull et al., 2007), especially in relation to the developmental phase of adolescence (Villalta et al., 2018). The theoretical and empirical contribution of the presented findings, focusing on the specific event COVID-19 and the adolescent population helps to more accurately describe the impact on psychological well-being and the need for support and intervention.

## Conclusion

The research hypotheses of the present study were largely supported by the analysis of the collected data and offer a significant contribution to support and complement what is already known on the topic of the traumatic effects of COVID-19 on adolescents and young adults and their relationship to emotional dysregulation. Further empirical confirmation of the definition of COVID-19 as a traumatic event for adolescents and young adults has been provided, as well as further empirical confirmation of the hypothesis, widely supported at the theoretical level, of a significant relationship between trauma and difficulties in emotion regulation in this population. In addition, the results of this study, at least in part, fill some gaps in the literature regarding the traumatic impact of COVID-19 on adolescents and the relationship between trauma and emotional dysregulation.

Further research is needed to examine the role of possible intervening variables in moderating the traumatic impact of specific events, such as personal positivity and hospitalisation.

In addition, our findings provide useful evidence for the development of intervention strategies that meet the needs of screening and supporting adolescents and young adults with regard to the traumatic effects of COVID-19 and related difficulties in emotion regulation, as well as prevention with regard to the possible consequences of stress, trauma, and emotional dysregulation in adolescence. Therefore, the collected data and this study are considered a renewed call not to underestimate the psychological impact of COVID-19, especially on the younger population, but to address it as an urgent and timely issue from both a restorative and preventive perspective.

However, several limitations of this study should be noted. First, no screening for preexisting PTSD or developmental trauma was performed. Second, the age of the sample included young adults, but given the diversity of the groups, no possible analysis was performed. Finally, no distinction was made between the direct and indirect effects of PTSD. In fact, the literature has shown that both direct and indirect effects of PTSD are important to analyze.

Direct exposure is strongly associated with PTSD, whereas indirect exposure, such as the unexpected death of a close family member or friend, can also cause psychological trauma symptoms. This distinction should be considered in further research.
